# Universal newborn hearing screening outcomes based on national health policy in Chiangrai Prachanukroh Hospital, Thailand

**DOI:** 10.1186/s12913-026-14654-4

**Published:** 2026-05-04

**Authors:** Krittipong Parangrit, Kanokwan Kulprachakarn, Suwicha Kaewsiri Isaradisaikul, Jutatip Sillabutra

**Affiliations:** 1 School of Health Sciences Research, Research Institute for Health Sciences, Chiang Mai University, Chiang Mai, 50200 Thailand; 2 Otolaryngology Unit, Chiangrai Prachanukroh Hospital, Chiang Rai, Thailand; 3 Department of Otolaryngology, Faculty of Medicine, Chiang Mai University, Chiang Mai, 50200 Thailand; 4 Department of Biostatistics, Faculty of Public Health, Mahidol University, Bangkok, Thailand

**Keywords:** Newborn hearing screening, Hearing loss, National health policy, Transient evoked otoacoustic emissions

## Abstract

**Background:**

Universal newborn hearing screening (UNHS) is essential for early identification of congenital hearing loss to decrease the adverse effects of a child’s speech and language development. Since 2021, Thailand has enforced newborn hearing screening, followed by the national health policy of the Ministry of Public Health. In order to meet this objective, the quality of these programs should be monitored using internationally recognized benchmarks. This study aimed to analyze the UNHS outcomes according to the national health policy and standard benchmarks.

**Methods:**

A retrospective study was conducted at Chiangrai Prachanukroh Hospital, a tertiary care hospital, from December 2021 to November 2022. All newborns delivered in a one-year period who underwent hearing screening were recruited. The coverage rate, rates of diagnostic hearing evaluation, and hearing rehabilitation were analyzed and compared between the well-baby newborns (WBN) and high-risk newborns (HRN).

**Results:**

Of 4,216 newborns delivered, 3,363 (79.8%) were WBN, and 853 (20.2%) were HRN. The screening coverage rates before 1 month of age were 94.6% and 72.2%, and referral rates were 34.7% and 34.1% for WBN and HRN, respectively, showing significant differences. The follow-up return rates were 51.9% and 45.7%, audiological diagnosis within 3 months of age were 11.8% and 10.5%, considering diagnosis within 6 months of age were 52.7% and 42.1% for WBN and HRN, respectively, showing without significant differences. Eight children were diagnosed with sensorineural hearing loss, three from the WBN group and five from the HRN group. One child, in the high-risk group, received bilateral hearing aids and speech therapy.

**Conclusion:**

UNHS serves as a critical initial measure for the early identification of hearing loss, paving the way for timely interventions. Despite the preliminary indicators not meeting the standard benchmarks, there is a clear necessity for systematically developing implementation protocols to enhance the program’s efficacy. The national health policy in Thailand should persist in its efforts, including providing resources such as manpower, money, and materials (3M’s), to ensure the program’s success.

## Background

Newborn hearing screening is crucial for the early detection of congenital hearing loss to mitigate its impact on delayed speech and language, cognitive impairment, academic development, and social-emotional impairment [1]. Early intervention within the first six months is vital due to the benefits of auditory neural plasticity for language skill development and rehabilitation [2]. Despite nearly 50% of newborns with congenital hearing loss showing no risk factors and being classified as well-baby newborns (WBN) [3, 4], universal newborn hearing screening (UNHS) remains essential to identify all newborns. According to the Early Hearing Detection and Intervention program, the Joint Committee on Infant Hearing (JCIH) 2007 recommends screening before 1 month, diagnostic confirmation of hearing loss before 3 months, and receiving appropriate intervention before 6 months of age [5]. The indicators based on JCIH 2019 (1-2-3 months of age) can be implemented after successfully meeting the benchmark indicators of the 1-3-6 goals [6]. Several countries have mandated screening, recognizing its healthcare importance, with hospitals adapting their methods to their context and resources, initially focusing on high-risk newborns (HRN). However, the majority of low- and middle-income countries face challenges due to limited resources, including equipment, human resources, budgetary support, and government policy mandates [7]. University (teaching) hospitals in Thailand, which are tertiary care centers, have achieved newborn screening coverage rates of 93.1% to 99.8% before hospital discharge. This high coverage is attributed to mandatory hospital policies, systematic data collection, multidisciplinary coordination, and strong family support [8–10]. Referral rates ranged from 5.3% to 14.5% due to ear canal debris, middle ear effusion, and NICU complications [8–10]. Low follow-up rate for newborns failing their initial hearing screening, ranging from 35.1% to 68.6%, due to a lack of awareness about the importance of hearing screening [7, 8, 10, 11]. Conversely, high follow-up rate, ranging from 81.4% to 98.4% were achieved through parent education knowledge and a hearing tracking system [9, 12–14]. The rate of audiological diagnosis before 3 months ranged from 20.3% to 99.3%, with subsequent intervention rates varying [9, 10, 12, 14]. Common barriers to timely diagnosis include loss to follow-up and the complex health conditions of NICU newborns [13, 15].

Since 2021, Thailand has enforced a newborn hearing screening program, directed by the Ministry of Public Health. The program prescribes otoacoustic emissions (OAE) tests for WBN and/or automated auditory brainstem response (AABR) for high-risk ones. It recommends screenings for all newborns by 1 month (with a goal of ≥ 95% coverage) and audiological evaluations by 6 months [16], with the government covering costs. Chiangrai Prachanukroh Hospital is a tertiary-level hospital in Northern Thailand with 800 beds, serving 1.2 million people. The hospital is responsible for about 47,000 referral cases from an extensive region including Chiang Rai and neighboring provinces like Phayao a year, facing this challenge acutely. It has otolaryngologists and audiologists equipped with physiological hearing devices like OAE screening, acoustic immittance measurement, and auditory evoked potential, as well as behavioral hearing devices such as visual reinforcement audiometry and conventional play audiometry, along with rehabilitation equipment. However, it currently lacks AABR due to a shortage of equipment. Chiangrai Prachanukroh Hospital, in line with the national health policy, has been performing UNHS using a two-stage transient evoked otoacoustic emissions (TEOAE) protocol since 2021. While most Thai studies focus solely on the screening stage for HRN, this study broadens the scope to include diagnosis and rehabilitation, offering vital insights into the effectiveness of early hearing detection and intervention in a tertiary-level hospital context. Our previous study demonstrated improvements in key performance indicators following the implementation of a new newborn hearing screening protocol compared to routine practice [17]. However, that study focused primarily on protocol-level efficacy. In contrast, the present study evaluates the overall outcomes of the UNHS under the national health policy, including screening, follow-up, diagnosis, and rehabilitation, as well as comparisons between WBN and HRN. Thailand currently applies the JCIH 2019 criteria to define high-risk factors for hearing loss. Therefore, this study aimed to analyze the preliminary outcomes of the UNHS following the national health policy. Currently, our country uses JCIH 2019 criteria for high-risk factors for hearing loss.

## Materials and methods

### Study design

This retrospective cohort study, conducted at Chiangrai Prachanukroh Hospital from December 2021 to November 2022, evaluated newborn hearing screening on WBN and HRN based on JCIH 2019 criteria [6]. At stage 1, all newborns received TEOAE screenings before discharge in the quiet room at the obstetric and pediatric wards for the first screening. Those failing the first screening had a repeat TEOAE test for the second screening by rotating nurses before closing hospital discharge to minimize referrals. The second screening period occurs approximately 24 h after the first screening. Full-time staff nurses with less than 6 months of experience conducted the screenings. Due to equipment limitations, AABR screenings were not used for HRN at risk of auditory neuropathy, highlighting resource constraints. At stage 2, newborns not passing the TEOAE were appointed to retest for the third screening at the ENT department. Subsequent failures led to further audiological assessment using ABR, ASSR, and tympanometry, with confirmed cases of hearing loss enrolled in early intervention within 6 months. The study included newborns delivered at Chiangrai Prachanukroh Hospital between December 2021 and November 2022. Exclusion criteria were set for deceased newborns, those transferred to another hospital before reaching 1 month of age. Congenital meatal atresia was excluded in HRN.

### Instrumentation

TEOAE is a nonlinear click stimulus that originates from the outer hair cells, transmits through the middle ear, and is measured in the external ear canal with a sensitive microphone. The click stimulus level typically ranges from 75 to 85 dB peSPL, with a frequency response between 1 and 4 kHz [18]. For hearing screening, the Sentiero device (Path Medical, Germany) is utilized. Results are automatically categorized as either ‘pass’ or ‘refer’ (fail). A ‘pass’ is defined by criteria including a stability of at least 80%, an artifact level not exceeding 20%, and a signal-to-noise ratio of at least 6 dB [10]. High-frequency middle ear tympanometry was performed on newborns under 6 months old using the MI34 middle ear diagnostic analyzer (Maico, Germany) [2]. For diagnostic ABR, the SmartEP system (Intelligent Hearing Systems, USA) was employed, utilizing click stimuli at intensities of 70, 50, and 30 dB nHL, with a stimulus rate of 19.3 clicks per second and a sweep count of 2000 in rarefaction polarity. This setup aims to identify wave V, crucial for estimating hearing levels. A hearing threshold at 30 dB nHL or below was considered indicative of normal hearing [2, 14].

### Statistical analysis

A chi-square test was performed to compare the proportion difference in the percentage between the two groups on categorical variables using SPSS version 21. Statistical significance was set to *p* < 0.05.

## Results

**Fig. 1:**
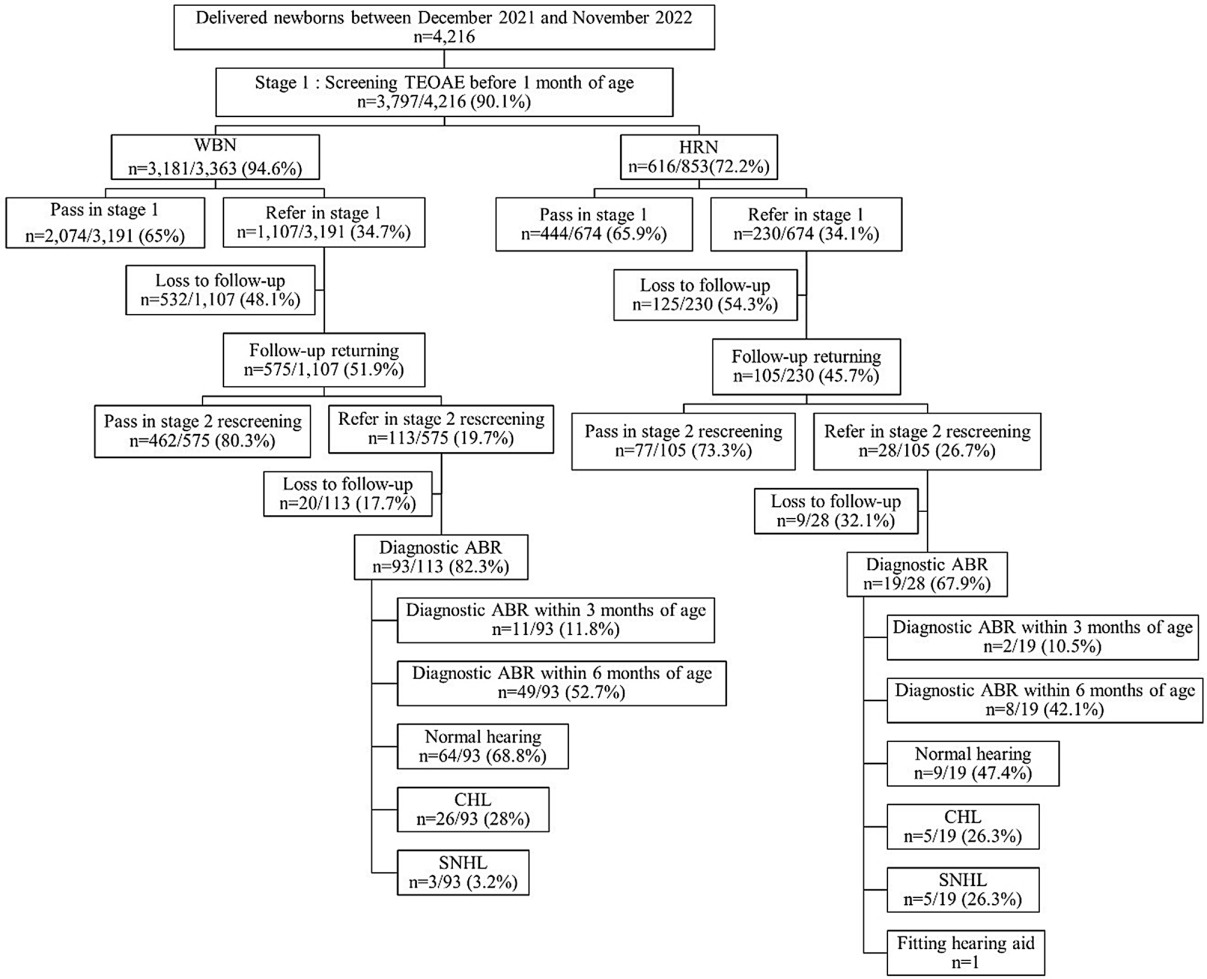
Flowchart of the universal newborn hearing screening program from 2021 to 2022. Abbreviation: TEOAE, transient evoked otoacoustic emissions; WBN, well-baby newborns; HRN, high-risk newborns; ABR, auditory brainstem response; CHL, conductive hearing loss; SNHL, sensori-neural hearing loss

During the one year from December 2021 to November 2022, Chiangrai Prachanukroh Hospital reported 4,216 births, with 3,797 newborns screened before 1 month. Initial screening referrals were 1,107 WBN and 230 HRN, reduced to 113 WBN and 28 HRN at rescreening. Among newborns who were referred after the initial screening, 575 WBN and 105 HRN returned for follow-up evaluation. Diagnostic ABR within 3 months showed 11 WBN and 2 HRN, and within 6 months, 49 WBN and 8 HRN. Due to follow-up loss, 20 WBN and 9 HRN were undiagnosed. ABR findings revealed 64 WBN with normal hearing, 26 with conductive hearing loss (CHL), and 3 with sensori-neural hearing loss (SNHL) without receiving hearing aids; among HRN, 9 had normal hearing, 5 had CHL or SNHL, with one receiving a hearing aid in the high-risk group. The flowchart of the UNHS program is shown in Fig. 1.

**Fig. 2:**
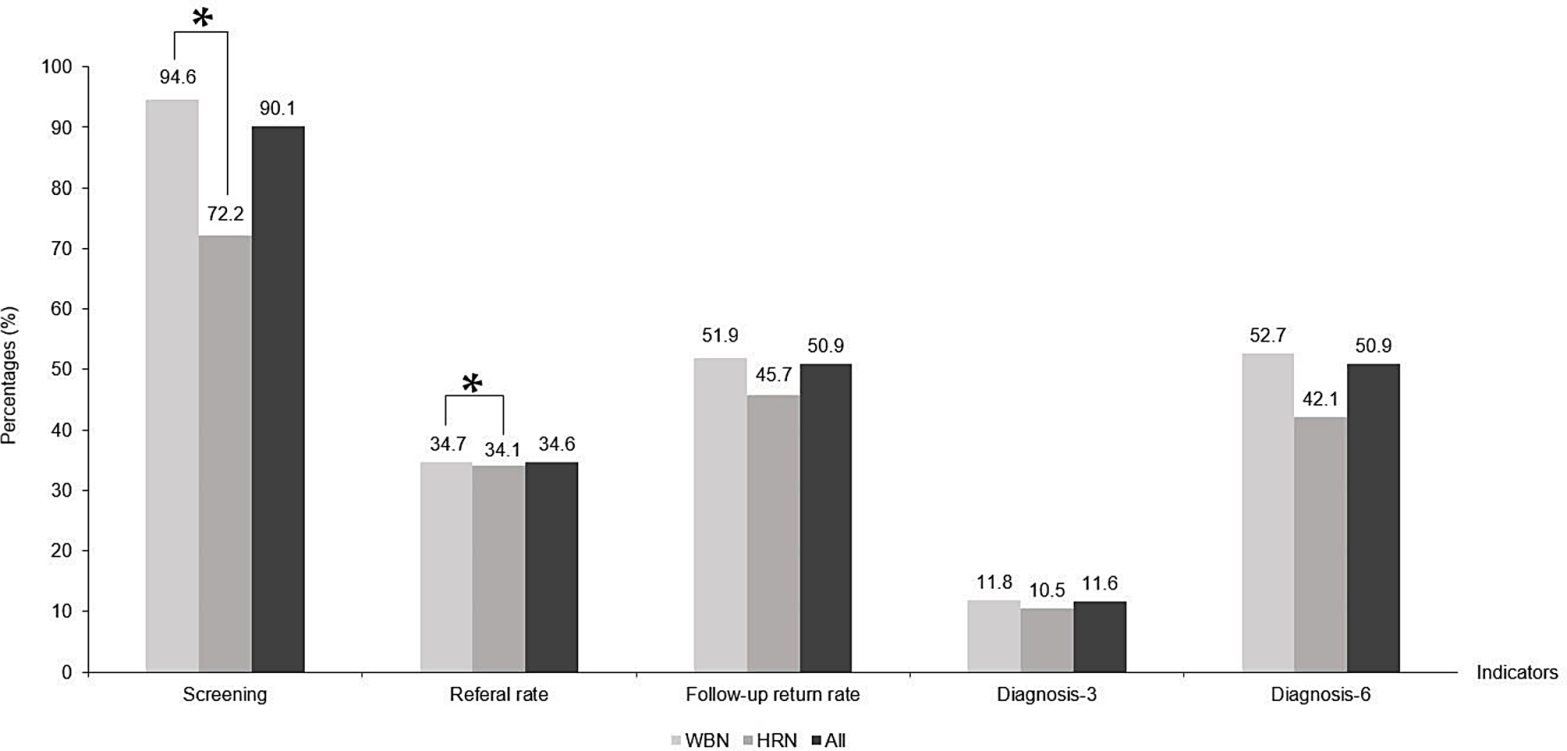
Results of newborn hearing screening program in Chiangrai Prachanukroh Hospital, Thailand from 2021 to 2022. Notes: * Significant at *p* < 0.05, compared between WBN and HRN, ** Results: Screening, screening ≤ 1 month of age; diagnosis-3 and − 6, Audiological diagnosis ≤ 3 and − 6 months of age. Abbreviation: WBN, well-baby newborns; HRN, high-risk newborns

According to the national guidelines of Thailand and JCIH benchmarks for the recommendation, the results are shown in Fig. 2. The screening coverage rates before 1 month of age were 94.6%, 72.2%, and 90.1% in WBN, HRN, and all newborns, respectively, with significant differences. The referral rates were 34.7%, 34.1%, and 34.6% in WBN, HRN, and all newborns, respectively with significant differences. The follow-up return rates were 51.9%, 45.7%, and 50.9% in WBN, HRN, and all newborns, respectively without significant differences. The audiological diagnosis before 3 months of age rates were 11.8%, 10.5%, and 11.6% in WBN, HRN, and all newborns, respectively without significant differences. When considering with the benchmarks of the Thailand guideline, the rates of audiological diagnosis before 6 months of age were observed to be 52.7%, 42.1%, and 50.9% in WBN, HRN, and all newborns, respectively without significant differences. It’s noteworthy that diagnostic rates experienced an uplift from the 3-month to the 6-month mark.

Table 1 shows the distribution of SNHL by severity across the studied newborns, which included 3 WBN and 5 HRN. The study found an equal number of cases of unilateral and bilateral hearing loss among these 8 children. The range extended from mild to profound degrees in both ears. According to the JCIH, risk factors for hearing loss include NICU stay for more than 5 days, congenital infections, birth asphyxia, and exposure to ototoxic medications such as aminoglycosides. In our cohort, specific risk factors identified among affected newborns included congenital syphilis (*n* = 2), birth asphyxia (*n* = 2), and aminoglycoside exposure (*n* = 1). However, this study did not perform a formal analysis of the association between these risk factors and hearing loss. Only one child received hearing aids and speech therapy at 18 months due to late diagnosis, while the other 7, with unilateral and mild hearing loss, were monitored but not given hearing aids. The study emphasizes the importance of auditory monitoring for at-risk newborns, suggesting visual reinforcement audiometry or conditioned play audiometry to catch late-onset or progressive hearing loss early. This underscores the critical need for UNHS because it is found in normal children as well. Among the newborns who underwent diagnostic evaluation, 31 cases were identified with CHL, primarily due to middle ear effusion. All of these cases received appropriate medical treatment.

**Table 1:** Risk factors, hearing loss severity, and interventions of 8 hearing loss children

Children No.	Risk factors	Degree of hearing loss		Intervention
		Right	Left	
1	No risk	Normal hearing	Mild SNHL	Watchful waiting and follow-up
2	No risk	Mild SNHL	Mild SNHL	Watchful waiting and follow-up
3	No risk	Mild SNHL	Mild SNHL	Watchful waiting and follow-up
4	-Admit NICU more than 5 days -Congenital syphilis	Normal hearing	Mild SNHL	Watchful waiting and follow-up
5	-Admit NICU more than 5 days -Birth asphyxia	Normal hearing	Moderate SNHL	Watchful waiting and follow-up
6	-Admit NICU more than 5 days -Congenital syphilis	Profound SNHL	Normal hearing	Watchful waiting and follow-up
7	-Admit NICU more than 5 days -Birth asphyxia	Mild SNHL	Mild SNHL	Watchful waiting and follow-up
8	-Admit NICU more than 5 days -Aminoglycoside	Moderate SNHL	Profound SNHL	Fittinghearing aids

## Discussion

UNHS is globally recognized for its critical role in early identification. This study followed Thai national policy and JCIH benchmarks, finding that HRN were screened later than WBN, mainly due to longer NICU stays or poor health conditions [12]. The screening coverage rate before 1 month was 90.1%, surpassing the 89.2% reported by Abdullah et al. in Malaysia, but falling short of the Thai national guideline and the JCIH benchmarks (≥ 95%) [19]. This shortfall was due to the newness of the UNHS program under the free national health policy from October 2021 onwards and competing responsibilities of staff nurses. In Southern China, a significant rise in screening rates from 95.2% to 99.5% between 2015 and 2020, as mandated by the Maternal and Infant Health Care Act, shows the potential for high coverage rates nationwide [20]. Limited resources, such as only two pieces of screening equipment across four wards, led to missed screenings and equipment breakdowns due to multiple screeners, particularly impeding HRN screening with the shortage AABR equipment. This reflects a broader issue in developing countries where advanced screening technology is costly [21]. Additionally, the process of recording hearing screening results was challenging, with some screeners failing to document outcomes properly. The lack of a dedicated multidisciplinary team further impeded progress. To improve coverage rates, it is crucial to institute a policy prioritizing the screening program within the hospital and adopt a multidisciplinary strategy [10, 22]. Developing a hearing screening database for systematic data collection and analysis will enhance program effectiveness [10, 21, 22]. Quality control should be implemented according to hospital resources and the hospital’s healthcare level. Chief executives and related agencies should support the operation in terms of personnel, budget, and screening equipment.

The referral rates significantly differed between WBN and HRN, with WBN having a slightly higher rate due to our hospital’s policy of reducing length of stay admissions, which led to an earlier discharge. Most WBN undergo screening a few hours post-birth, increasing referral rates due to vernix caseosa or debris in the ear canal and amniotic fluid in the middle ear, leading to high false positives [7, 23]. Our referral rate of 34.6% was lower than the 47% reported by Bezuidenhout et al. [7] but still above the JCIH benchmark of ≤ 4%. This inconsistency might be due to inexperienced staff and high noise levels during testing [12]. A two-tier screening protocol, starting with OAE and followed by AABR, can reduce referral rates [6, 20]. Screenings after the first 48 h of life can also significantly reduce referral rates [10, 24]. Middle ear effusions in WBN typically resolve by 4.8 months [4], but this issue may persist in HRN with conditions like cleft palate or Down’s syndrome. Improving screening quality requires enhancing staff proficiency, conducting screenings in a low-noise environment (e.g., < 65 dBA), and ensuring proper probe placement [25].

The follow-up return rate between WBN and HRN showed no significant difference, with WBN slightly higher, but the overall follow-up rate was 50.9%, well below the JCIH benchmark of ≥ 95%. This high loss to follow-up reduces program effectiveness, risking undetected congenital hearing loss. Parangrit K [26] found that personal commitments, forgotten appointments, transportation issues, underestimating follow-up importance, COVID-19 concerns, the newborn’s illness, and lack of counseling were reasons for missed follow-ups. Socioeconomic factors also play a role. Improving follow-up rates can be achieved by implementing a newborn database and hearing tracking system, educating parents in the postpartum ward, providing phone reminders, and establishing a dedicated multidisciplinary team.

The audiological diagnosis rates within 3 months were 11.8% for WBN and 10.5% for HRN, with no significant differences. By 6 months according to the national guideline of Thailand, the rates were 52.7% for WBN and 42.1% for HRN, still below the JCIH benchmark (≥ 90%). Delays in diagnosis were due to high referral rates leading to long appointment wait times, a shortage of audiologists at Chiang Rai Prachanukroh Hospital, the need for multiple evaluations complicated by newborns’ sleep states during ABR testing, postponement for ill newborns, and parental scheduling conflicts. Reducing waiting times can be addressed by managing the high referral rate and implementing a ‘fast track system’ [9]. Increasing the number of audiologists will also help provide timely evaluations [10]. Adherence to pre-ABR testing instructions by parents can minimize repeat visits. The prevalence of congenital hearing loss among the newborns in both groups was 0.9% (0.9% in WBN and 1.2% in HRN groups, respectively), which was higher than in previous study (0.5%) [3, 4]. A year after policy implementation, several limitations were identified. The high number of newborns failing initial screening and not returning for follow-up makes it difficult to ascertain the false positive rate. Only TEOAE was used for screening, which is less sensitive to auditory neuropathy compared to ABR, affecting NICU newborns due to the lack of AABR equipment. Lastly, as this is a retrospective study, manual data collection may result in missing or incomplete information.

## Conclusion

UNHS is critical for early hearing loss detection, allowing for timely interventions that greatly benefit children’s development. Without screening, hearing loss may remain undiagnosed until adverse effects on speech and language development emerge at school age. Although the quality indicators did not meet the standard benchmarks initially, there is a clear path forward. The multidisciplinary team and establishing a centralized database for efficient tracking and follow-up of screening results are key to this enhancement. Additionally, the success of the newborn hearing screening program in Thailand hinges on sustained and expanded national health policy, supported by adequate resources (manpower, money, materials—3 M’s). Future research should focus on developing and refining protocols that enhance the newborn hearing screening program’s efficacy, ensuring quality control and ultimately improving the child’s development.

## Data Availability

No datasets were generated or analysed during the current study.

## Abbreviations

ABR: Auditory brainstem response
CHL: Conductive hearing loss
HRN: High-risk newborn
NICU: Neonatal intensive care unit
SNHL: Sensori-neural hearing loss
TEOAE: Transient evoked otoacoustic emissions
WBN: Well-baby newborns

## References

[1] Turan Z, Baş N. Evaluation of the effectiveness of newborn hearing screening program: A center in Turkey. Int j early child spec. 2019;11(2):141–53.

[2] Colella-Santos MF, Hein TA, de Souza GL, do, Amaral MI, Casali RL. Newborn hearing screening and early diagnostic in the NICU. Biomed Res Int. 2014;2014:845308. 10.1155/2014/845308 PMC4066868 24999481

[3] Mishra G, Sharma Y, Mehta K, Patel G. Efficacy of Distortion Product Oto-Acoustic Emission (OAE)/Auditory Brainstem Evoked Response (ABR) Protocols in Universal Neonatal Hearing Screening and Detecting Hearing Loss in Children < 2 Years of Age. Indian J Otolaryngol Head Neck Surg. 2013;65(2):105–10. 24427548 10.1007/s12070-012-0553-2 PMC3649019

[4] White KR, Forsman I, Eichwald J, Munoz K. The evolution of early hearing detection and intervention programs in the United States. Semin Perinatol. 2010;34(2):170–9. 20207267 10.1053/j.semperi.2009.12.009

[5] American Academy of Pediatrics JCIH. Year 2007 position statement: Principles and guidelines for early hearing detection and intervention programs. Pediatrics. 2007;120(4):898–921. 17908777 10.1542/peds.2007-2333

[6] American Academy of Pediatrics JCIH. Year 2019 position statement: Principles and guidelines for early hearing detection and intervention programs. Pediatric. 2019;4(2):1–44. 10.1542/peds.2007-2333 17908777

[7] Bezuidenhout JK, Khoza-Shangase K, De Maayer T, Strehlau R. Outcomes of newborn hearing screening at an academic secondary level hospital in Johannesburg, South Africa. S Afr J Commun Disord. 2021;68(1):e1–8. 33567828 10.4102/sajcd.v68i1.741 PMC7876983

[8] Thongsrinuch P, Junthong S. Prevalence of early hearing detection and intervention newborn in Vajira hospital. Vajira Med J. 2021;65:39–52.

[9] Chouyboonchum T, Chamchoi P, Chaikhamrongkul T, Isarangura S, Tiravanitchakul R. Universal Newborn Hearing Screening and Incidence of Hearing Loss in Ramathibodi Hospital: A 5 Years Experience (2014 to 2018). RMJ. 2022;45(4):25–34.

[10] Khaimook W, Suwanno R, Dindamrongkul R, Intusoma U. An Early Hearing Detection and Intervention Program in Songklanagarind Hospital. J Health Sci Med Res. 2022;40(5):551–9.

[11] Alanazi AA. Referral and Lost to System Rates of Two Newborn Hearing Screening Programs in Saudi Arabia. Int J Neonatal Screen. 2020;6(3):50. 33123632 10.3390/ijns6030050 PMC7570083

[12] Li PC, Chen WI, Huang CM, Liu CJ, Chang HW, Lin HC. Comparison of Newborn Hearing Screening in Well-Baby Nursery and NICU: A Study Applied to Reduce Referral Rate in NICU. PLoS ONE. 2016;11(3):e0152028. 27023324 10.1371/journal.pone.0152028 PMC4811549

[13] Fukunaga I, Kobayashi T, Hirose K. Screening Newborns for Hearing Loss under Full Public Funding, Kochi, Japan -Differences in the Screening Results between Premature Neonates and Healthy Newborns. JMA J. 2022;5(2):263–7. 35611218 10.31662/jmaj.2021-0203 PMC9090546

[14] Ciorba A, Hatzopoulos S, Corazzi V, Cogliandolo C, Aimoni C, Bianchini C, et al. Newborn hearing screening at the Neonatal Intensive Care Unit and Auditory Brainstem Maturation in preterm infants. Int J Pediatr Otorhinolaryngol. 2019;123:110–5. 31096068 10.1016/j.ijporl.2019.05.004

[15] Upadhyay K, Gupta V, Singh S, Bhatia R, Lohith BR, Reddy NM, et al. Outcome of Universal Neonatal Hearing Screening Programme at a Tertiary Care Centre: A Prospective Study. Indian J Otolaryngol Head Neck Surg. 2022;74(Suppl 3):3813–8. 36742795 10.1007/s12070-021-02628-3 PMC9895671

[16] Yimtae K, Potaporn M, Kaewsiri S. The committee of newborn hearing screening guidelines of Thailand. 1st ed. Bangkok: Off-set; 2019 Apr.

[17] Parangrit K, Sillabutra J, Isaradisaikul SK, Kulprachakarn K. Comparative study of the efficacy between new and routine newborn hearing screening protocols in public hospital, Thailand. J Matern Fetal Neonatal Med. 2025;38(1):2531150. 40701817 10.1080/14767058.2025.2531150

[18] Kosmidou P, Tzifas S, Lygeros S, Danielides G, Nikolopoulos T, Dimitriou G, et al. Newborn Hearing Screening: Analysing the Effectiveness of Early Detection of Neonatal Hearing Loss in a Hospital in Greece. Cureus. 2021;13(11):e19807. 34956791 10.7759/cureus.19807 PMC8693701

[19] Abdullah A, Hazim MY, Almyzan A, Jamilah AG, Roslin S, Ann MT, et al. Newborn hearing screening: experience in a Malaysian hospital. Singap Med J. 2006;47(1):60–4. 16397723

[20] Wang Y, Cheng C, Li C. Newborn hearing loss in the south of China: a cross-sectional study. J Int Med Res. 2021;49(12):3000605211062448. 34861130 10.1177/03000605211062448 PMC8647265

[21] Friderichs N, Swanepoel D, Hall JW 3. Efficacy of a community-based infant hearing screening program utilizing existing clinic personnel in Western Cape, South Africa. Int J Pediatr Otorhinolaryngol. 2012;76(4):552–9. 22326208 10.1016/j.ijporl.2012.01.015

[22] Wasser J, Ari-Even Roth D, Herzberg O, Lerner-Geva L, Rubin L. Assessing and monitoring the impact of the national newborn hearing screening program in Israel. Isr J Health Policy Res. 2019;8(1):30. 30857547 10.1186/s13584-019-0296-6 PMC6410489

[23] De Luca LM, Malesci R, Gallus R, Melis A, Palmas S, Degni E, et al. Audiological risk factors, referral rates and dropouts: 9 years of universal newborn hearing screening in North Sardinia. Child (Basel). 2022;9(9). 10.3390/children9091362 PMC9497641 36138671

[24] Mackey AR, Busse AML, Del Vecchio V, Maki-Torkko E, Uhlen IM. Protocol and programme factors associated with referral and loss to follow-up from newborn hearing screening: a systematic review. BMC Pediatr. 2022;22(1):473. 35932008 10.1186/s12887-022-03218-0 PMC9354382

[25] Salina H, Abdullah A, Mukari SZ, Azmi MT. Effects of background noise on recording of portable transient-evoked otoacoustic emission in newborn hearing screening. Eur Arch Otorhinolaryngol. 2010;267(4):495–9. 19727788 10.1007/s00405-009-1080-y

[26] Parangrit K. Factors affecting newborn hearing screening follow-up at Otolaryngology department,Chiangrai Prachanukroh Hospital. CMJ. 2022;14(3).

